# Ring-Opening Polymerization of Trimethylene Carbonate with Phosphazene Organocatalyst

**DOI:** 10.3390/polym15030720

**Published:** 2023-01-31

**Authors:** Jianglin Zhu, Xiaoming Luo, Xin Li

**Affiliations:** Southern Marine Science and Engineering Guangdong Laboratory (Zhanjiang), Zhanjiang 524000, China

**Keywords:** PTMC, *t*-BuP_4_, controlled/living nature, end functionality

## Abstract

Aliphatic polycarbonate (APC) compounds are an important class of biodegradable materials with excellent biocompatibility, good biodegradability, and low toxicity, and the study of these compounds and their modification products aims to obtain biodegradable materials with better performance. In this context, the ring-opening polymerization (ROP) of trimethylene carbonate (TMC) from a low nucleophilic organic superbase of phosphazene (*t*-BuP_4_) as a catalyst and benzyl alcohol (BnOH) as an initiator at room temperature was carefully studied to prepare poly(trimethylene carbonate) (PTMC) which is one of the most studied APC. ^1^H NMR and SEC measurements clearly demonstrate the presence of a benzyloxy group at the *α*-terminus of the obtained PTMC homopolymers while investigation of the polymerization kinetics confirms the controlled/living nature of *t*-BuP_4_-catalyzed ROP of TMC. On the basis of this, the block copolymerization of TMC and *δ*-valerolactone (VL)/*ε*-caprolactone (CL) was successfully carried out to give PTMC-*b*-PCL and PTMC-*b*-PVL copolymers. Furthermore, PTMC with terminal functionality was also prepared with the organocatalytic ROP of TMC through functional initiators. We believe that the present ROP system is a robust, highly efficient, and practical strategy for producing excellent biocompatible and biodegradable PTMC-based materials.

## 1. Introduction

Biodegradable polymeric materials have important applications in many biomedical fields, such as drug delivery, tissue engineering, and surgical medicine, and their research is receiving increasing attention [1,2,3,4]. Aliphatic polycarbonate (APC) compounds are an important class of biodegradable materials with excellent biocompatibility, good biodegradability, and low toxicity, and the study of these compounds and their modification products aims to obtain biodegradable materials with better performance [5,6,7,8]. In the past decades, great efforts have been made to develop functional materials based on APCs by controlling their composition, structure, molecular weight, dispersity, and functionality. Among them, amorphous poly(trimethylene carbonate) (PTMC) with a glass transition temperature of −17 °C, represents the most studied for its potential application in biomedical fields [9,10,11,12].

In general, PTMC was produced by ring-opening polymerization (ROP) of the cyclic monomer of trimethylene carbonate with metal-based catalysts, such as Sn(II)2-ethylhexanoate (SnOct_2_) and aluminum isopropoxide Al(O-*i*-Pr)_3_ [13]. However, due to the inherent disadvantages of the widely accepted “coordination-insert” polymerization mechanism for metal-based ROP catalysts, the residues resulting from the metallic catalyst seem difficult to remove from the final product, as they appear as impurities, and thus limit the further application of PTMC in biomedical fields, where even very small amounts of catalyst residues cannot be accepted [14,15,16,17]. Although enzymes have been used to produce PTMC instead of metal catalysts, it generally takes longer to produce polymers with a molecular weight below 10^4^ g/mol, which would not be suitable for the applications [18,19,20]. Therefore, it is still highly attractive to develop new catalysts for the ROP of TMC to produce PTMC with high efficiency and controllability.

Organocatalysts, which have been extensively surveyed since the pioneering work of Hedrick and Waymouth over the last two decades, have also been used prominently to proceed the ROP of TMC, and they typically include organic acids (e.g., sulfonic and phosphoric acids and their derivatives) and organic bases (e.g., guanidine, amidine, and carbene) [21,22,23,24,25]. Aiming at how acidity affected catalytic activity, Hedrick and co-workers used molecular modeling to calculate the energy based on the reaction mechanism, and the results showed that catalysis is indeed dependent on both acid strength and the ability of the conjugate base to act as a hydrogen-bond acceptor, suggesting the superiority of bifunctional activation [26].

Among various organocatalysts, phosphazenes, representing a type of nitrogen-phosphor hybrid organobase with low nucleophilicity, have been widely used for the ROP of epoxides, cyclic lactams, lactones, siloxanes, and methacrylates [27,28,29]. Bulk ROP of TMC with a less basic organocatalyst 2-*tert*-butylimino-2-diethylamino-1,3-dimethylperhydro-1,3,2-di-azaphosphorine (BEMP) with an alcohol initiator produced PTMC with high molecular weight at 100 °C (*M*_n_ = 45,800 g/mol, *Ð* = 1.49), while polymer with lower molecular weight and lower dispersity were obtained at lower temperatures (60 °C), but polymerization control is lost at higher temperatures leading to larger dispersities [30,31]. However, compared to an ocean of publications focusing on the ROP of cyclic lactones, the ROP of carbonate catalyzed by phosphazene receives less attention. In particular, the strongest basicity and non-nucleophilicity of phosphazene *t*-BuP_4_ (^MeCN^p*K*a is 42.6) not only transform protic compounds into nucleophilic initiating species by deprotonation or activation of weak initiators but also guarantee that the polymerization process is not influenced by catalysts due to nucleophilicity [32]. Based on the above discussions, in this study, we perform (a) ROP of TMC catalyzed by *t*-BuP_4_ and the details of the polymerization kinetics, (b) the preparation of block copolymers composed of PTMC and poly(*δ*-valerolactone) (PVL)/poly(*ε*-caprolactone) (PCL). We envision that it can afford a versatile and robust way to make PTMC with *t*-BuP_4_.

## 2. Materials and Methods

### 2.1. Materials

TMC from Aladdin (Shanghai, China) was purified by recrystallizing twice from anhydrous tetrahydrofuran (THF) and stored at −10 °C in a glove box [33]. CL and VL from Aldrich (Shanghai, China) were stirred overnight with calcium hydride (CaH_2_), distilled under reduced pressure, and stored in a glove box. Benzyl alcohol (BnOH) from Aladdin was purified by refluxing sodium under nitrogen for two hours and then distilling under reduced pressure. Azeotropic distillation using anhydrous toluene (TOL) was selected to purify methoxy poly(ethylene glycol) (*m*PEG, *M*_n_ = 2.0 × 10^3^ g/mol, Aldrich) due to the hydrophilic nature of PEG. Anhydrous TOL and THF (Aladdin) were obtained by distillation from the sodium/benzophenone mixture on the basis of the color change of the mixture (purple indicates the absence of water) and stored in a glove box. Other reagents from Aldrich were used as received.

### 2.2. Methods

Characterization by proton nuclear magnetic resonance (^1^H NMR) was performed on a Bruker AV400 NMR (Bruker, Billerica, MA, USA) spectrometer using deuterated chloroform (CDCl_3_) as the solvent, to which tetramethylsilane (TMS) was added as an internal standard. A Waters size exclusion chromatography (SEC, Waters, Milford, MA, USA) system equipped with a model 510 pump, a model 410 differential refractive index (RI) detector and a Waters 2487 UV detector operating at a wavelength of 254 nm was used to measure the number average molecular weight (*M*_n_) and dispersity (*Ð*) of the isolated polymers at 35 °C with an eluent rate of 1.0 mL/min (HPLC-THF, Aladdin), while a series of monodisperse polystyrene samples were used as standards to calibrate the molecular weight tested. The samples had a concentration of ~5 mg/mL, and a 400 nm hydrophobic PTFE membrane was used to clean the samples prior to detection. A Bruker-Autoflex III Smartbeam MALDI-TOF MS machine (Bruker, Germany) operated at an accelerating voltage of 25 kV (reflector mode) was used to measure the absolute molecular weight and end-functionality of the obtained polymers (HPLC THF was used as solvent; a mixture of 50 μL of 10 mg/mL polymer, 50 μL of 15 mg/mL 2,5-dihydroxybenzoic acid matrix, and 10 μL of 4.0 mg/mL sodium iodide cationic agent was deposited on the sample platform and then dried for testing). Differential scanning calorimetry (DSC) measurement was conducted on a NETZSCH DSC 200F3 machine (Netzsch, Berlin, Germany) at a scan rate of 10 °C/min under a flowing nitrogen atmosphere. The sample was rapidly heated to 150 °C and held for 10 min to remove thermal history and then cooled to −50 °C at a rate of 10 °C/min. Subsequently, the sample was reheated to 150 °C at the same rate, where the glass transition temperature (*T*_g_) was taken as centered around the transition of the curve. The thermogravimetric analysis (TGA) test was carried out on a TA SDT Q600 instrument (TA, New Castle, DE, USA) in a nitrogen atmosphere with a heating rate of 10 °C/min in the range of 25–600 °C. The samples used for the thermal test were previously dried under vacuum at 80 °C. 

### 2.3. Polymerization

**Figure 1:**
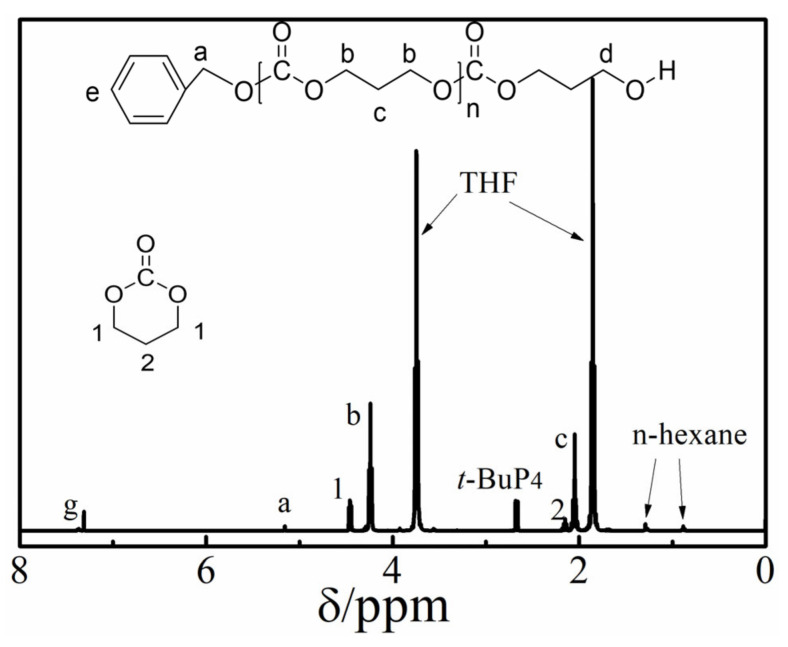
^1^H NMR spectrum of the reaction mixture (Table 1 and S3, 30 min.) in CDCl_3_.

The details of a typical preparation for the ROP of TMC using BnOH as an initiator and *t*-BuP_4_ as a catalyst are as follows: TMC (0.51 g, 5.0 mmol, 50 equiv), THF (2 mL), and BnOH (10.40 μL, 0.1 mmol, 1.0 equiv) were first placed in a dried Shrek flask fitted with a magnet stirrer in a glove box. Subsequently, *t*-BuP_4_ (50 μL, 0.05 mmol, 1.0 equiv in hexane) was added to the flask to start the polymerization. In order to measure the evolution of molecular weight and *Ð* value on the monomer conversion, a small amount of the reaction mixture was taken for ^1^H NMR analysis and SEC measurement for the designated time. Finally, the reaction mixture was poured into a large excess of cold methanol to precipitate the polymer after the designated time, and a white solid was obtained after drying in a vacuum. The polymerizations of TMC under other conditions and the polymerizations of PCVL or PCL were carried out in a similar way (Table 1). PTMC (Yield: 90%, *M*_n,SEC_ = 5500 g/mol, *Ð* = 1.18, Figure 1), ^1^H NMR (400 MHz, CDCl_3_, ppm): 2.03 (2H, −COOCH_2_C*H*_2_CH_2_O−), 3.63 (2H, −COO(CH_2_)_2_C*H*_2_OH), 4.22 (4H, −COOC*H*_2_CH_2_C*H*_2_O−), 5.10 (2H, PhC*H*_2_O−), 7.33 (5H, aromatic); PCL (Yield: 90%, *M*_n,SEC_ = 5200 g/mol, *Ð* = 1.30). 1.33 (2H, −CO(CH_2_)_2_C*H*_2_(CH_2_)_2_O−), 1.63 (4H, −COCH_2_C*H*_2_CH_2_*CH*_2_CH_2_O−), 2.28 (2H, −COC*H*_2_(CH_2_)_4_O−), 3.63 (2H, −CO(CH_2_)_4_C*H*_2_OH), 4.05 (2H, −CO(CH_2_)_4_C*H*_2_O−), 5.10 (2H, PhC*H*_2_O−), 7.32 (5H, aromatic); PVL (Yield: 97%, *M*_n,SEC_ = 5200 g/mol, *Ð* = 1.20), 1.65 (4H, −COCH_2_(C*H*_2_)_2_CH_2_O−), 2.32 (−COC*H*_2_(CH_2_)_3_O−), 3.65 (2H, −CO(CH_2_)_3_C*H*_2_OH), 4.05 (2H, −CO(CH_2_)_3_C*H*_2_O−), 5.10 (2H, PhC*H*_2_O−), 7.34 (5H, aromatic).

The details of a typical preparation for the block copolymerization of TMC and CL using BnOH as an initiator and *t*-BuP_4_ as a catalyst are as follows: TMC (0.30 g, 3.0 mmol, 30 equiv), BnOH (10.40 μL, 0.1 mmol, 1.0 equiv), and THF (3 mL) were first placed in a dried Shrek flask fitted with a magnet stirrer in a glove box. Subsequently, *t*-BuP_4_ (100 μL, 0.1 mmol, 1.0 equiv) was added to the flask to start the polymerization. After allowing the TMC polymerization to proceed for 4 h, an aliquot was taken from the flask and quenched to determine the molecular properties of the PTMC block, and then the polymerization of the second block was started by the addition of CL (0.34 g, 3.0 mmol, 30 equiv). The polymerization was quenched by the addition of 0.1 mL of acetic acid after an additional period of 4 h, and the reaction mixture was precipitated in a large excess of cold methanol to give white solid PTMC-*b*-PCL. (Yield 80%). ^1^H NMR (Table 2, 400 MHz, CDCl_3_, ppm): 1.35 (2H, −CO(CH_2_)_2_C*H_2_*(CH_2_)_2_O−), 1.65 (4H, −COCH_2_C*H_2_*CH_2_*CH_2_*CH_2_O−, 4H, −COCH_2_C*H*_2_*CH_2_*CH_2_O−), 2.32 (2H, −COC*H_2_*(CH_2_)_4_O−, 2H, −COC*H*_2_(CH_2_)_3_O−), 3.63 (2H, −CO(CH_2_)_4_C*H*_2_OH, 2H, −CO(CH_2_)_3_C*H*_2_OH), 4.07 (2H, −CO(CH_2_)_4_C*H*_2_O−, 2H, −COC*H*_2_(CH_2_)_3_OH), 5.10 (2H, PhC*H*_2_O−), 7.32 (5H, aromatic). The copolymerization of TMC and VL was performed in a similar process to give PTMC-*b*-PVL (yield 68%). ^1^H NMR (Table 2, 400 MHz, CDCl_3_, ppm): 1.34 (2H, −CO(CH_2_)_2_C*H*_2_(CH_2_)_2_O−), 1.63 (4H, −COCH_2_C*H*_2_CH_2_*CH*_2_CH_2_O−), 2.04 (2H, −COOCH_2_C*H*_2_CH_2_O−), 2.30 (2H, −COC*H*_2_(CH_2_)_4_O−), 3.65 (2H, −CO(CH_2_)_4_C*H*_2_OH), 4.06 (2H, −CO(CH_2_)_4_C*H*_2_O−), 4.12 (4H, −COOC*H*_2_CH_2_C*H*_2_O−), 5.10 (2H, PhC*H*_2_O−), 7.32 (5H, aromatic).

## 3. Results and Discussion

### 3.1. Ring Opening Polymerization of TMC by t-BuP_4_

**Scheme 1:**
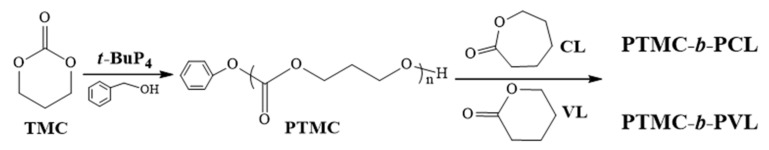
General scheme for the ring-opening polymerization of TMC using benzyl alcohols as initiators and *t*-BuP_4_ as catalyst, as well as the synthesis of block copolymers.

First, *t*-BuP_4_ was used to catalyze the ROP of TMC with BnOH as the initiator and THF as the solvent at room temperature (Scheme 1). Figure 1 shows the ^1^H NMR spectrum of S3 (Table 1) over 30 min. The signals attributed to the methylene protons of the PTMC backbone and smaller signals due to the methylene protons of the benzyloxy group are clearly observed, indicating that *t*-BuP_4_ and BnOH separately played the role of catalyst and initiator in the ROP system. In addition, the integration of the methylene peak attributed to BnOH is equivalent to the area of the methylene peak adjacent to the terminal hydroxyl group at the *ω*-chain end, implying good chain-end fidelity. It is worth noting that the main side reaction in the cyclic carbonate ROP, the formation of ether bonds by decarboxylation, did not occur in the catalytic system according to ^1^H NMR analysis. In fact, some monomer is still present in the reaction mixture from Figure 1, and the monomer conversion is about 80% according to the ^1^H NMR calculation (monomer conversion reaches ~95% after 8 h).

Notably, the number-average molecular weight (*M*_n,NMR_) calculated according to ^1^H NMR spectra and that based on the conversion rate and the initial monomer/initiator feed ratio maintain good consistency (Table 1). The SEC trace of the obtained PTMC is monomodal, while the *Ð* values are below 1.30. These results demonstrate that the room-temperature ROP of TMC catalyzed by *t*-BuP_4_ has good control over molecular weight and dispersity. PVL and PCL were also prepared using *t*-BuP_4_ as a catalyst and BnOH as an initiator. We found that the rate of consumption of CL is lower than that of VL (Table 1), which is consistent with other ROP situations involving VL and CL [34,35,36,37,38,39].

### 3.2. Controlled/Living Manner of the ROP of PTMC by t-BuP_4_

**Figure 2:**
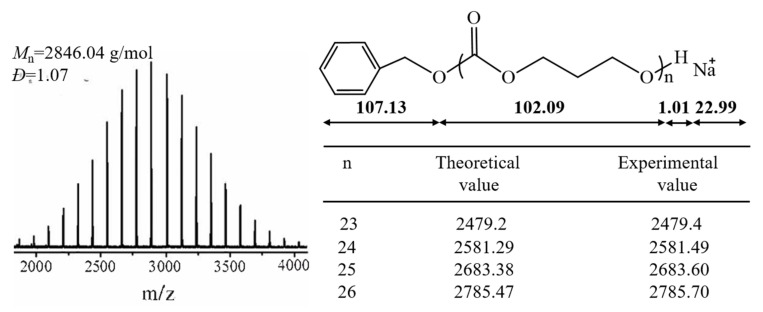
MALDI-TOF MS spectra of the obtained PTMC. The theoretical values were calculated using the following equation: 107.13 + n × 102.09 + 1.01 + 22.99, where n is the degree of polymerization and the mass values correspond to the segments and end groups comprising the PTMC chain as shown in the scheme.

**Figure 3:**
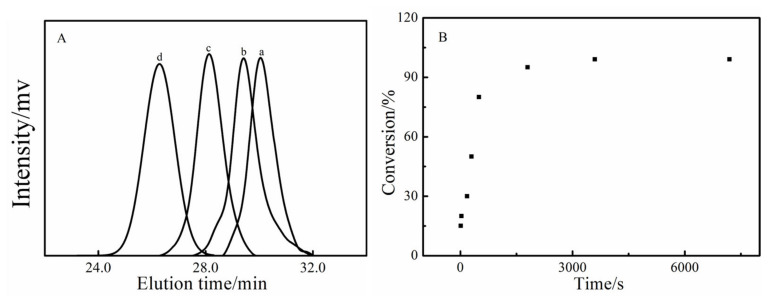
(**A**) SEC curves of PTMC obtained with different initial monomer/initiator ratios: a 10, b 30, c 50, d 100, (**B**) Dependence of monomer conversion on reaction time.

In order to further confirm that the *t*-BuP_4_-catalyzed ROP of TMC was initiated by BnOH, we performed MALDI-TOF MS analysis (Figure 2). From the figure it can be seen that there is only a series of single peaks consistent with the molecular weight of PTMC calculated with BnO- and hydroxyl as chain ends. This result indicates that the *t*-BuP_4_-catalyzed ROP of TMC with the initiator of BnOH proceeds in a controlled or living manner with negligible side reactions (e.g., backbiting, transesterification). Figure 3A displays the SEC curves of the isolated PTMC with different monomer/initiator ratios (30 to 100). It is obvious that the *M*_n,SEC_ of the obtained PTMC increases with increasing monomer to initiator ratio, which is also evidence for controllable polymerization. Figure 3B clearly shows that monomer conversion increases with reaction time, and the monomer conversion achieved 80% at about 10 min, indicating the high reactivity of *t*-BuP_4_-catalyzed ROP of TMC (which is important to the controllability).

**Figure 4:**
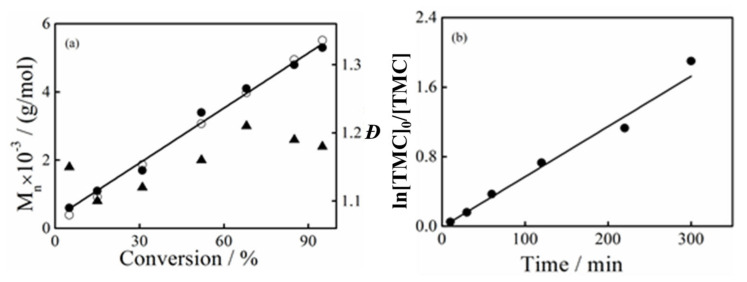
(**a**) Dependence of *M*_n,SEC_ (○), *M*_n,theo_ (●) and *Ð* (▲) of the obtained PTMC on the conversion of TMC and (**b**) kinetic plot for the *t*-BuP_4_ catalyzed ROP of TMC initiated with BnOH.

After that, we focused on the polymerization kinetics of the *t*-BuP_4_-catalyzed ROP of TMC. As shown in Figure 4a, *M*_n,SEC_ (○) increases linearly with increasing monomer conversion, consistent with *M*_n,theo_. (●) while the values of *Ð* remains stable (~1.15) during the polymerization process. Figure 4b shows that the monomer conversion and polymerization time represent the typical kinetic nature of the first-order polymerization, which not only indicates the stable consumption of monomer during the ROP but also further confirms the living feature of the *t*-BuP_4_-catalyzed ROP of TMC [37,38,39].

**Figure 5:**
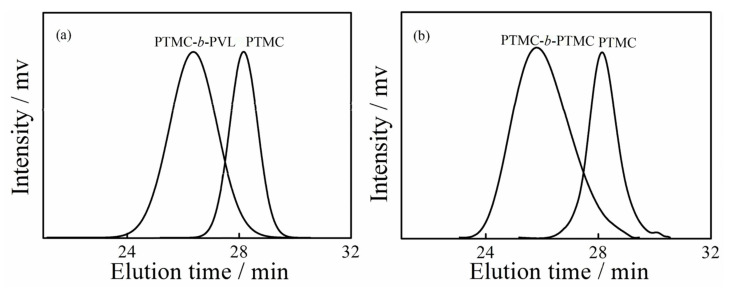
SEC curves of (**a**) PTMC-*b*-PVL and (**b**) PTMC-*b*-PCL copolymers.

Chain extension experiments are also commonly used to demonstrate active polymerization. Figure 5 shows the SEC curves of the chain extension experiments with PTMC comprising a hydroxyl chain end as the macroinitiator (Table 2, runs 3 and 4). Subsequently, we added 40 equiv of VL to the reaction mixture containing PTMC (*M*_n,SEC_ = 2600, *Ð* = 1.13) macroinitiator and thus a PTMC-*b*-PVL block copolymer was obtained (*M*_n,SEC_ = 7500 g/mol, *Ð* = 1.24, Figure 5a). Compared with the PTMC polymer, the SEC curve of the block polymer has shifted to the high molecular weight region while maintaining a low *Ð* value, indicating that the chain extension polymerization has good controllability. PTMC-*b*-PCL was prepared in a similar way, and the molecular weight of the polymer increased from 2500 g/mol (PTMC homopolymer) to 8000 g/mol and *Ð* increased from 1.12 to 1.32 (Figure 5b). These results demonstrate that the PTMC chain ends retain their active or living character for further polymerization, thereby providing a method for preparing biodegradable PTMC-based block copolymers.

### 3.3. A Plausible Reaction Mechanism

**Scheme 2:**
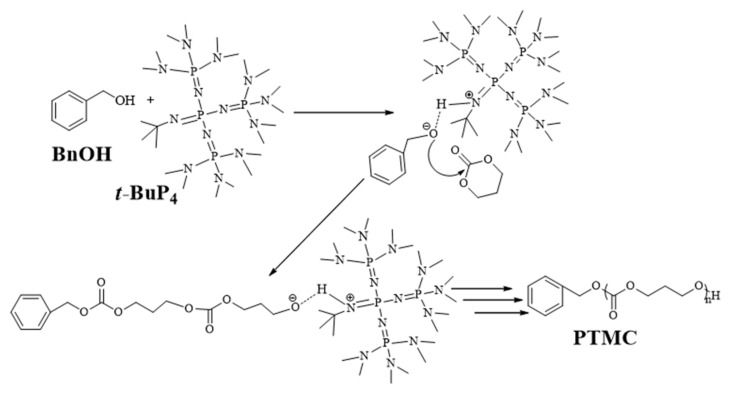
A possible initiator/chain-end mechanism for *t*-BuP_4-_catalyzed ROP of TMC.

A possible initiator/chain-end mechanism is proposed in Scheme 2 (see also other works on the anionic ROP of TMC through the initiator/chain-end mechanism). An alkoxy active center was generated by the hydrogen bond of *t*-BuP_4_ with the initiator alcohol [40,41,42]. Then, alkoxy active center attacks the carbonyl of TMC subsequently with ring opening reaction to form the alkoxy active growth chain end. Then, the active chain end continues to carry out the ring opening of TMC to form PTMC homopolymer.

### 3.4. Preparation of Functional PTMC

**Figure 6:**
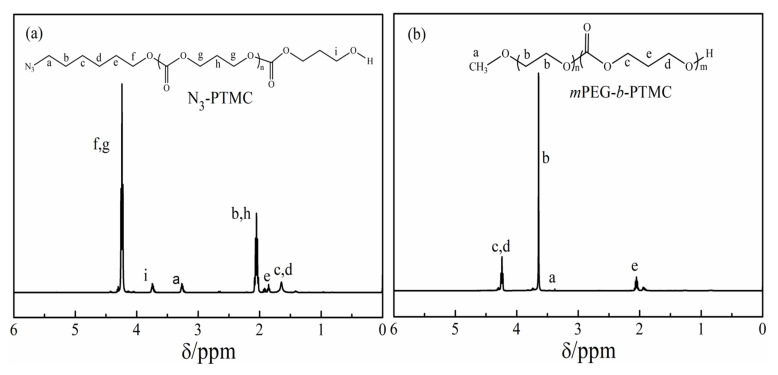
^1^H NMR spectra of PTMC initiated by (**a**) 6-azido-1-hexanol (AHA) (N_3_-PTMC) and (**b**) methoxy poly(ethylene glycol) (*m*PEG-*b*-PTMC).

In order to further explore the possible applications of this catalytic system, we attempted to synthesize end-functionalized PTMC with functionalized initiators. We employed 6-azide-1-hexanol (AHA) as an initiator to prepare PTMC-based polymers with azido at one end, and polymers with block, branch, and other topological structures could be easily assembled using the highly efficient azido-alkyne click reaction with the synthesized alkyne-terminal polymers. From the ^1^H NMR spectrum (Figure 6a), we can clearly see a signal attributed to methylene hydrogen adjacent to azido, and the molecular weight *M*_n,NMR_ (1960 g/mol) calculated from the ^1^H NMR spectrum was consistent with the theoretical calculation value *M*_n,theo_ (2148 g/mol). PEG was one of the most commonly applied commercial products in light of its prominent biocompatibility, antifouling, and so-called “stealth” character [43,44]. Thus, a macroinitiator, *m*PEG, was also used to initiate the ROP of TMC with a *t*-BuP_4_ catalyst, and an amphiphilic block copolymer, PEG-*b*-PTMC, was successfully synthesized (Figure 6b), which may find application in intracellular imaging and drug delivery systems. These results confirm that this catalytic system has a wide range of applications in the preparation of functionalized polycarbonate-based materials.

### 3.5. Thermal Properties of the Obtained PTMC

**Figure 7:**
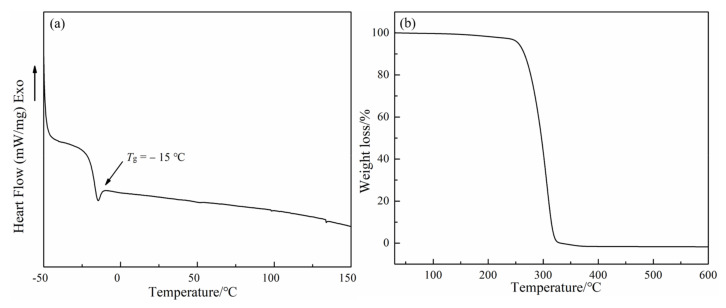
The DSC (**a**) and TGA (**b**) curves of PTMC.

Finally, we focused on the thermal properties of the PTMC prepared by the *t*-BuP_4_-catalyzed ROP of TMC to extend this study. Figure 7 shows the DSC (a) and TGA (b) curves of the obtained PTMC. As we know, PTMC is amorphous with a glass transition temperature (*T*_g_) of around −17 °C [45]. The homopolymer of PTMC is a white powder when the molecular weight is higher than 3 × 10^3^ g/mol; below this, it appears as a waxy solid. The DSC measurement was chosen to examine the *T*_g_ of the isolated PTMC. Figure 7a clearly shows that the *T*_g_ of the tested PTMC (5 × 10^3^ g/mol) is around −15 °C, which is close to the literature, while the difference may be due to the different molecular weights of the polymers. This result also confirms the pure structure of the PTMC synthesized by the *t*-BuP_4_-catalyzed ROP of TMC. Figure 7b shows the TGA curve of the PTMC produced by the *t*-BuP_4_-catalyzed ROP of TMC. The degradation temperature of PTMC is similar to that reported [45], and there is only one stage of degradation, which also confirms the homopolymer of PTMC.

## 4. Conclusions

In summary, *t*-BuP_4_ organic superbase was investigated to exhibit its catalytic performance on the ring-opening polymerization of carbonate (TMC) at room temperature, and the results demonstrated that the polymerizations were in a controlled or living manner, allowing isolated polymers with designed molecular weight and narrow dispersity with *t*-BuP_4_ as the catalyst and BnOH as the initiator. Well-defined PTMC-*b*-PVL and PTMC-*b*-PCL were also prepared through the chain-extension method. In addition, end-functionalized PTMCs with azido group and mPEG-*b*-PTMC copolymer were also produced using 6-azido-1-hexanol (AHA) and methoxy poly(ethylene glycol) (mPEG) as functional initiators. *t*-BuP_4_ could be removed from the crude product with high feasibility just by precipitation to give PTMC with fewer catalyst residues. DSC and TGA characterizations also confirm the expected structure of PTMC produced by the *t*-BuP_4_-catalyzed ROP of TMC. In conclusion, we have demonstrated that *t*-BuP_4_ is an excellent catalyst in the preparation of PTMC-based functional polymers.

## Figures and Tables

**Table 1:** Summarized results of the ROP of TMC, CL, and VL using *t*-BuP_4_ as a catalyst and BnOH as an initiator. ^a^

Samples	Monomer	[M]^0^/[I]^0 b^	Time (h)	Conv. (%) ^c^	*M*_n,NMR_ × 10^−4^ (g/mol) ^c^	*M*_n,theo_ × 10^−4^ (g/mol) ^d^	*M*_n,SEC_ × 10^−4^ (g/mol) ^e^	*Ð* ^e^
S1	TMC	10	1	99	0.10	0.11	0.13	1.07
S2	TMC	30	4	97	0.30	0.33	0.29	1.07
S3	TMC	50	8	95	0.48	0.54	0.55	1.18
S4	TMC	100	10	94	1.10	1.04	1.22	1.19
S5	TMC	200	17	95	1.90	2.07	2.16	1.26
S6	TMC	500	24	85	3.52	4.85	4.32	1.30
S7	VL	30	1	99	0.30	0.30	0.41	1.18
S8	VL	50	2	98	0.42	0.51	0.52	1.20
S9	CL	100	4	98	0.94	1.08	0.98	1.25
S10	CL	50	4	98	0.45	0.49	0.52	1.55

^a^ Reaction temperature is 25 °C, THF as solvent; ^b^ Initial molar ratio of monomer to initiator; ^c^ Determined by ^1^H NMR; ^d^ Calculated from ([M]_0_/[I]_0_) × conv. × (MW of TMC) + (MW of initiator); ^e^ Determined by SEC.

**Table 2:** Synthesis of end-functionalized and block copolymers of PTMC. ^a^

Run	Monomer	Initiator	Conv.(%) ^b^	*M*_n,NMR_ (g/mol) ^b^	*M*_n,theo_ (g/mol) ^c^	*M*_n,SEC_ (g/mol) ^d^	*Ð* ^d^
1	TMC	AHA	97	1960	2148	2500	1.07
2	TMC	*m*PEG	98	5960	6000	6900	1.17
3	VL	PTMC ^e^	99	--	--	7500	1.24
4	CL	PTMC ^e^	90	--	--	8000	1.32

^a^ Reaction temperature was 25 °C with THF as solvent; ^b^ determined by ^1^H NMR; ^c^ calculated from ([M]_0_/[I]_0_) × conv. × (MW of TMC) + (MW of initiator); ^d^ determined by SEC; ^e^ PTMC macroinitiator.

## Data Availability

The data could be supported when required.
